# Manufacturing Polymer Model of Anatomical Structures with Increased Accuracy Using CAx and AM Systems for Planning Orthopedic Procedures

**DOI:** 10.3390/polym14112236

**Published:** 2022-05-31

**Authors:** Paweł Turek, Damian Filip, Łukasz Przeszłowski, Artur Łazorko, Grzegorz Budzik, Sławomir Snela, Mariusz Oleksy, Jarosław Jabłoński, Jarosław Sęp, Katarzyna Bulanda, Sławomir Wolski, Andrzej Paszkiewicz

**Affiliations:** 1 Faculty of Mechanical Engineering and Aeronautics, Rzeszów University of Technology, 35-959 Rzeszów, Poland; lprzeszl@prz.edu.pl (Ł.P.); gbudzik@prz.edu.pl (G.B.); jsztmiop@prz.edu.pl (J.S.); 2 Institute of Medical Science, University of Rzeszów, 35-959 Rzeszów, Poland; damian.a.filip@gmail.com (D.F.); ssnela@poczta.onet.pl (S.S.); ortopik@poczta.onet.pl (J.J.); 3 Orthopedics and Traumatology Department, University Hospital, 35-301 Rzeszów, Poland; 4 Department of Orthopedics and Traumatology, Holy Family Hospital, 36-060 Głogów Małopolski, Poland; lazorko@wp.pl; 5 Faculty of Chemistry, Rzeszów University of Technology, 35-959 Rzeszów, Poland; molek@prz.edu.pl (M.O.); k.bulanda@prz.edu.pl (K.B.); 6 Faculty of Mathematics and Applied Physics, Rzeszów University of Technology, 35-959 Rzeszów, Poland; wolan@prz.edu.pl; 7 Department of Complex Systems, Faculty of Electrical and Computer Engineering, Rzeszów University of Technology, 35-959 Rzeszów, Poland; andrzejp@prz.edu.pl

**Keywords:** polymer model, additive manufacturing, reverse engineering, Industry 4.0, accuracy anatomical structures, medical engineering, IT tools

## Abstract

Currently, medicine uses typical industrial structure techniques, including reverse engineering, data processing, 3D-CAD modeling, 3D printing, and coordinate measurement techniques. Taking this into account, one can notice the applications of procedures used in the aviation or automotive industries based on the structure of Industry 4.0 in the planning of operations and the production of medical models with high geometric accuracy. The procedure presented in the publication shortens the processing time of tomographic data and increases the reconstruction accuracy within the hip and knee joints. The procedure allows for the partial removal of metallic artifacts from the diagnostic image. Additionally, numerical models of anatomical structures, implants, and bone cement were developed in more detail by averaging the values of local segmentation thresholds. Before the model manufacturing process, additional tests of the PLA material were conducted in terms of its strength and thermal properties. Their goal was to select the appropriate type of PLA material for manufacturing models of anatomical structures. The numerical models were divided into parts before being manufactured using the Fused Filament Fabrication technique. The use of the modifier made it possible to change the density, type of filling, number of counters, and the type of supporting structure. These treatments allowed us to reduce costs and production time and increase the accuracy of the printout. The accuracy of the manufactured model geometry was verified using the MCA-II measuring arm with the MMDx100 laser head and surface roughness using a 3D Talyscan 150 profilometer. Using the procedure, a decrease in geometric deviations and amplitude parameters of the surface roughness were noticed. The models based on the presented approach allowed for detailed and meticulous treatment planning.

## 1. Introduction

**Figure 1:**
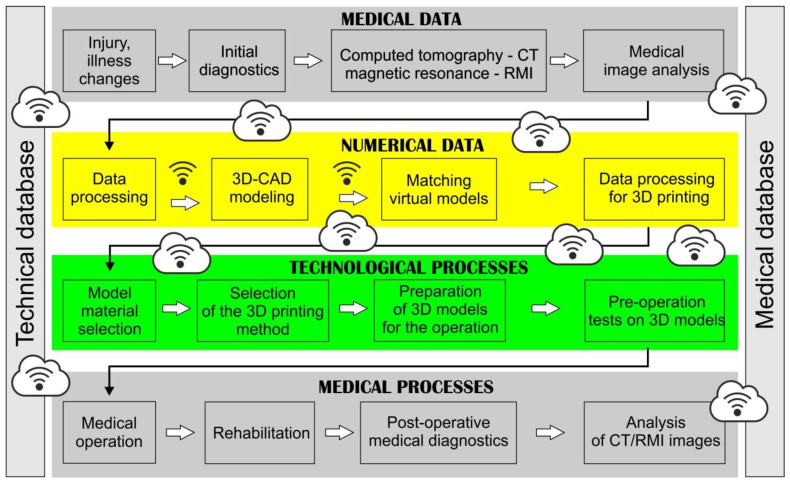
Diagram of integration of medical and technological systems using numerical data flow based on Industry 4.0 tools.

Reverse engineering is a process that enables the reconstruction of the geometry of an already existing object [1,2]. It covers activities related to data acquisition, reconstruction of the geometry of measured objects, and the transformation of the obtained data into a form that allows for their use in Computer-Aided Design (CAD) systems. Currently, reverse engineering is used in many areas, including the aviation industry [2,3,4] and architecture [2]. It is also often used in medicine, for example, to reconstruct the geometry of anatomical structures [5,6]. The integration of diagnostic and production systems in medical engineering is possible, thanks to modern IT tools based on the Industry 4.0 structure. In this case, database systems based on cloud disk spaces, wireless data transfer, wired data transfer systems, and granting data authorizations depending on the place in the system hierarchy are used. A diagram of the integration of medical and technological systems using the elements of the Industry 4.0 structure is shown in Figure 1.

Designing anatomical models, surgical templates, or implants for planning or direct implementation of a surgical procedure is a complex task that requires a great deal of experience, engineering knowledge, and a good understanding of anatomy. Errors may occur at each stage of reconstructing the geometry of the anatomical structure or the implant [7,8,9,10]. They can significantly affect the quality of the performed surgery. Therefore, it is crucial to identify and correct errors at each stage of the process. The accuracy of mapping the geometry of anatomical structures is mainly influenced by the stage of digitization [11,12,13,14] and the processing of volumetric data [9,10,15]. Then, the obtained data is transformed into a three-dimensional model. At this stage, the most crucial role is played by the segmentation process and the process of reconstructing the geometry into a three-dimensional form [10,16,17]. The accuracy of the geometry of the anatomical structure is also influenced by the selection of the manufacturing method [18,19,20]. The ready model can be made using subtractive techniques [21,22]. However, due to anatomical models’ complex structures and unique geometries, additive methods are most often used for their production [19]. Currently, on the market, there is a wide variety of devices and methods of shaping models based on incremental methods [18]. The differences in their functioning occur mainly in hardening subsequent layers and the type of material used. Each device used in additive technologies has specific characteristics and requirements for working conditions (a type of material, process temperature, and model finishing treatment) [23]. Despite the variety and availability of many methods, none of them dominate in medical applications, mainly due to the different properties of the materials used and the requirements for ready-made models [19,24].

The most significant number of reports on the use of printed three-dimensional models is presented in certain specialties, such as maxillofacial surgery and dentistry (58.3%) [25,26,27,28,29], as well as orthopedics (23.7%) [30,31,32]. Hip and knee arthroplasty is currently one of the most frequently performed orthopedic surgeries [33]. According to the data, the number of procedures performed in global terms increases year by year. These are scheduled strategies to improve the comfort and quality of patients’ lives. In most cases, the results are: the reduction or elimination of pain, restoration of the hip or knee joint functionality, and improvement of the patients’ quality of life. Unfortunately, similar to any invasive procedure, arthroplasty carries the risk of complications that may destroy the excellent result of the surgery and, instead of improving the patient’s quality of life, they reduce it. The most common complications include postoperative lower-limb-length difference, improper positioning of prosthesis components and their displacement, luxations, periprosthetic fractures, and septic or aseptic loosening. These complications significantly reduce the patient’s quality of life and entail problems for the patient and the entire healthcare system by extending the period and costs of hospitalization and making it impossible to return to professional activity, generating fees for the whole of society.

Printed bone models enable operators to prepare for surgery better than when just based on radiological diagnostics alone. They allow a better understanding of the existing pathology before and during surgery [34,35,36]. In addition, the models provide an almost unlimited possibility to practice the selected surgical technique to optimize the treatment and avoid errors during the procedure itself. However, continuous improvement of the process is required to increase the accuracy of the reconstruction of the geometry of bone structures. This is especially important when the diagnostic image shows metallic structures within bone tissues essential for the surgeon. To date, there is no literature on developing a systematic procedure for the reconstruction of geometry and the production of bone structures within the hip and knee joint using additive techniques. Thanks to the application of the presented procedure, it will be possible to reproduce the 3D models more accurately, which will allow for increasing the precision of fitting the template or the finished prosthesis during the procedure. Thus, it will be possible to avoid some of the complications.

## 2. Materials and Methods

**Figure 2:**
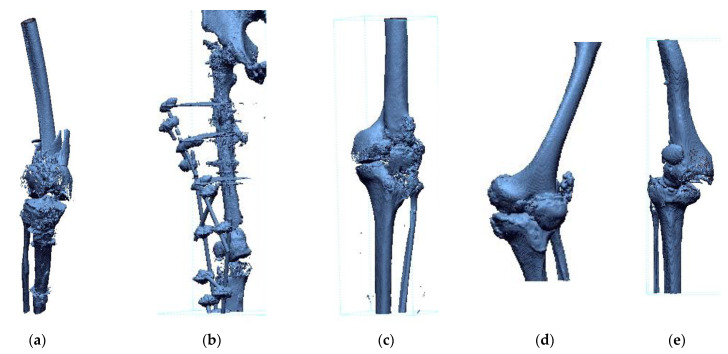
Cases of patients suffering from pathology of the knee joint: (**a**) Patient No. 1; (**b**) Patient No. 2; (**c**) Patient No. 3; (**d**) Patient No. 4; and (**e**) Patient No. 5.

**Figure 3:**
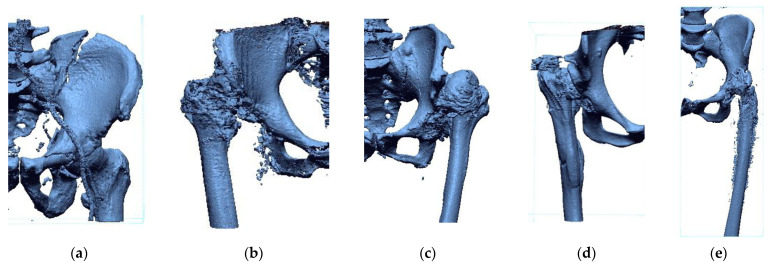
Cases of patients suffering from pathology of the hip joint: (**a**) Patient No. 6; (**b**) Patient No. 7; (**c**) Patient No. 8; (**d**) Patient No. 9; and (**e**) Patient No. 10.

Studies were conducted on a group of 10 patients from 2017–2020. In the study group, 5 cases related to the pathology of the knee joint (Figure 2) and 5 cases related to the pathology of the hip joint (Figure 3).

**Figure 4:**
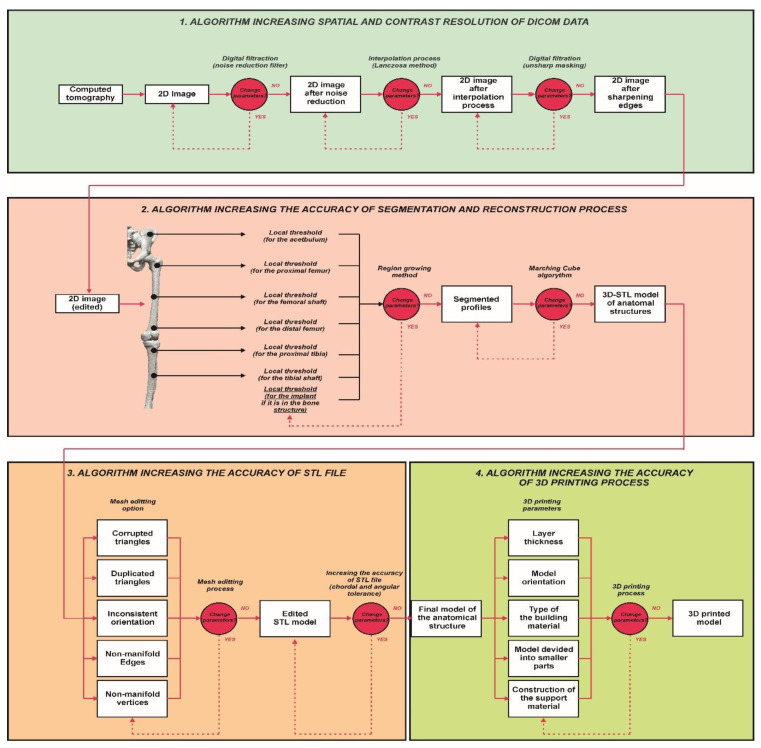
Procedure applied in the research.

There were four women and six men in the group of 10 patients. The oldest patient was 75 years old, and the youngest was 31 years old. The selection of patients concerned non-standard pathologies, which were a significant problem in selecting the surgical technique and implants. Based on the research performed on the presented group of patients, a procedure was developed and patented that allowed us to shorten the time of data processing and increase the accuracy of modeling and production of surgical templates using 3D-printing techniques to increase the precision of procedures in the hip and knee joints (Figure 4). This publication describes the procedure on the example of 4 randomly selected patients out of 10 included in the research process.

### 2.1. Digital Data Processing

**Figure 5:**
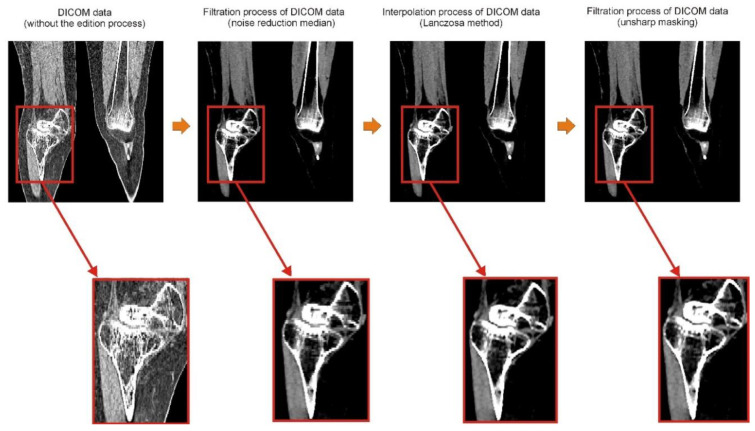
Digital filtration process and data interpolation improving the diagnostic quality of the DICOM data within the area of the knee joint at the stage of the design process.

The Provincial Clinical Hospital No. 2 in Rzeszów performs a standard scanning protocol within the hip and knee joint area. Diagnostics were performed using a Discovery CT750HD multi-slice tomograph (GE Healthcare, Chicago, IL, USA). The obtained Digital Imaging and Communications in Medicine (DICOM) data had some limitations. Therefore, to minimize them, it was undertaken to develop a procedure at the stage of numerical data processing, which allows for increasing the spatial and contrast resolution of DICOM data. Digital data processing was used to improve the image quality, which, in the first stage, involved removing noise from the images (minimum noise reduction filter). A new digitally processed image was obtained by applying the filtration process. The filtering of digital images in the spatial domain was obtained using the convolution operation (multiplication of two frequency-domain transforms, i.e., the image and the filter transform, which is equivalent to the convolution of the image with the filter in the spatial domain). The convolution operation computed a new pixel value for the image based on the values of the adjacent pixels. The next stage of the procedure was to increase the spatial resolution of the image by using the interpolation method [37,38]. Image interpolation consists of determining additional pixels and their value based on the intensity of adjacent pixels. This is often referred to as image scaling, image resampling, or image resizing. Interpolation methods calculate missing pixel values from the data provided by the original image. To date, the disadvantage of this solution was the significant increase in the size of volumetric data, which required more memory and resources for rendering. Due to the substantial development of computerized systems, this problem no longer exists. The interpolation methods differ in determining additional pixels and their value. However, the resulting image will always be slightly blurred regardless of the interpolation method, so the filtration process was used at the last data processing stage. It sharpened the boundary between the bone structure and soft tissue (unsharpmasking filter). The entire data processing procedure is presented in Figure 5.

**Figure 6:**
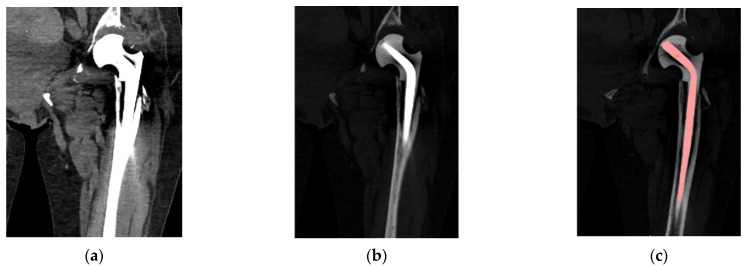
Result of the application of the procedure in the case of the occurrence of metallic structures: (**a**) Image with the DICOM data not processed; (**b**) Image with the DICOM data processed; and (**c**) Marking the part of an implant in the segmentation process.

The digital data processing procedure was also used in the case of the occurrence of metallic structures within the analyzed bone tissues (Figure 6a,b). Thanks to the implementation of the shown procedure, it is possible to separate the implant from the bone tissue more efficiently (Figure 6c).

### 2.2. Segmentation and Reconstruction of the Geometry

**Figure 7:**
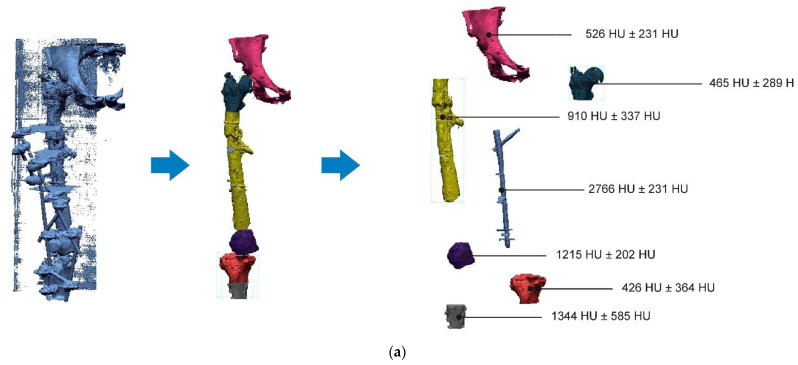

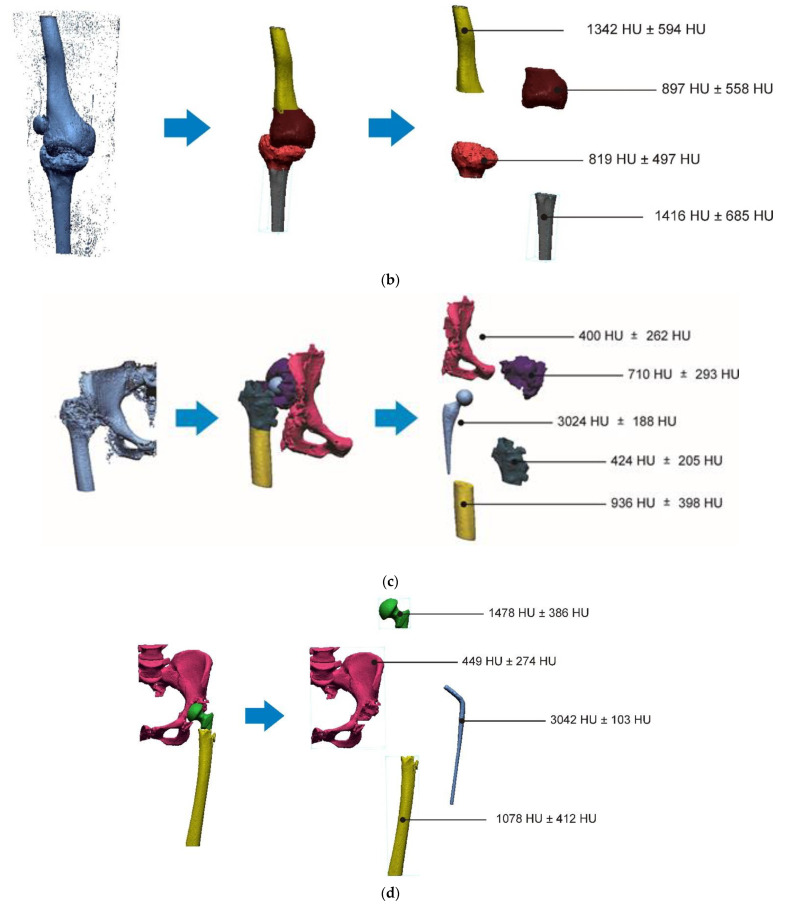
Improving the segmentation process with the application of the local threshold as exemplified by: (**a**) Bone structures within the area of the hip joint and the knee joint with the implant—Patient No. 2; (**b**) Bone structures within the area of the knee joint—Patient No. 5; (**c**) Bone structures within the area of the hip joint with the implant and the bone cement—Patient No. 7; and (**d**) Bone structures within the area of the hip joint with the implant—Patient No. 10.

A segmentation process was performed on the digitally processed image. Thanks to the data processing process, the extraction of the bone structure was significantly accelerated with the simultaneous increase in the accuracy of geometry reconstruction. The segmentation process extracted an interesting operator anatomical structure from the entire data set. In segmenting the structures within the knee and hip joints, the region growing method was used. The local thresholding method was used to define the boundary conditions for this method, against which the bone structure was extracted. This procedure aimed to increase segmentation accuracy within the extracted bone structure by selecting an individual threshold in the designated area. Based on the averaged results obtained from the 10 analyzed patients, local segmentation thresholds for the bone structures within the hip and knee joints were developed. They were defined based on information on the averaged values of units in the HU scale assigned to pixels representing a given bone structure. The segmentation threshold values for metallic structures (Figure 7a,c,d) and the bone cement (Figure 7c) were determined. The effects of the implementation of the procedure in the context of planning a surgical procedure were presented for four selected patients of the proposed group (Figure 7a–d).

**Figure 8:**
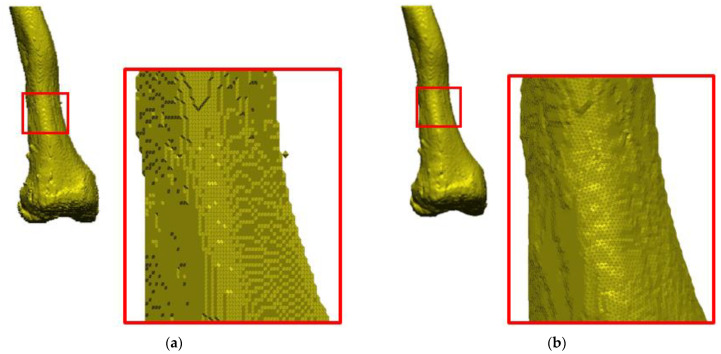
Process of editing the faceted surface: (**a**) Prior to editing and (**b**) following editing.

The isosurface method was used to illustrate the spatial model to be later printed. It is one of the indirect surface methods, and it is based on the marching cube algorithm [39,40]. This algorithm works so that the space is divided into a series of cubes that can span one or more voxels. Then, the nodes of individual designated cubes are checked in terms of the defined iso-value. Depending on whether the node’s value was greater or less, polygons corresponding to the isosurface passing between these points were inserted in the place of the cube. There are 256 cube orientations regarding the surface. However, 15 unique canonical orientations can be distinguished. For simplifying the algorithm, others can be obtained by their rotation, mirroring, and inversion of the normal ones. The algorithm has its drawbacks, so at the surface treatment stage, it is necessary to edit the faceted surface (Figure 8a).

### 2.3. Increasing the Accuracy of the STL Model

After reconstructing the geometry, an additional edition of the faceted surface (mesh of triangles) was performed (Figure 8b), which consisted of verifying that:

- Normal vectors are inverted;
- Gaps between the triangles appear;
- Surface distortion;
- Lack of the entire surface or its selected fragments;
- Overlapping and intersecting triangles;
- No common edge;
- Addition of free triangles with no bounds.

The most common error in the reconstructed geometry of anatomical structures concerns the direction of normal vectors, which should be directed outwards for all triangles. This is a prerequisite for the accurate description of the faceted surface. This error appeared on single fragments of the triangle mesh, selected areas, or the entire surface [41]. In saving the model to a format representing a triangle mesh, there were also mapping inaccuracies related to angular deviation or chord deviation [42,43]. Reducing the deviation of the chord and the angular deviation allows for an increase in the accuracy of mapping curvilinear surfaces. However, it should be remembered that if you want to reproduce the geometry as accurately as possible using a triangle mesh, it should be high densities. However, it is related to the enlargement of the file saved in the *.stl format. The size of the triangle mesh and its density on selected surfaces is closely related to the additive manufacturing method, which will be chosen at the following stages of the anatomical model. Densifying the mesh of triangles is sometimes necessary because the geometry of the anatomical models of the hip or knee joint structures is complex and consists mainly of curvilinear surfaces. In addition, the procedure includes the option of removing inclusions that are invisible during the export of data, which were created in the process of segmentation of the anatomical structure. They should be removed to increase the accuracy of the mapping of the model made in manufacturing processes.

### 2.4. Tests of PLA-Based Polymer Materials and the Manufacturing of Anatomical Structures

The Prusa i3 MK3 device (*Prusa* *Research*, *Prague*, *Czech Republic*) was used in the 3D-printing process. This printer works in the Fused Filament Fabrication (FFF) [44,45] additive technology. In this method, the principle of applying a layered thermoplastic material is practically the same as in the Fused Deposition Modeling (FDM) method. The main criteria for selecting the device were its availability, low price, and the possibility of using various thermoplastic polymers in this technique [46]. The extrusion temperature did not exceed 300 °C. As mentioned earlier, the preparation of the process for the device occurred in the PrusaSlicer software environment. All models were printed from a PLA thermoplastic polymer [47,48,49]. It is one of the most widely suitable polymeric materials in the additive FFF method. The cost of good-quality material per kilogram does not exceed several dozen dollars. This material is characterized by good strength properties and allows printing in an open working space. Due to the low-processing shrinkage, more excellent geometric stability is maintained than in thermoplastic materials. In addition, this material does not cause difficulties during the printing process. This material is highly workable and has a relatively low extrusion temperature. 

Before the model manufacturing process, additional tests of the PLA material were conducted in terms of its strength and thermal properties. Their goal was to select the appropriate type of PLA material in the process of manufacturing models of anatomical structures. The research was focused on obtaining PLA composites. The following raw materials were used for the tests following the literature recommendations [50,51]: polylactide (UltraPLA, Noctuo, hydroxyapatite marked as Hap, and hydroxyapatite surface-modified with poly(ethylene glycol) marked as HAp mod. To produce composites, PLA was mixed with unmodified and modified hydroxyapatite in specified proportions to obtain materials with 0.5%, 1%, and 1.5% for each filler, as shown in Table 1.

**Figure 9:**
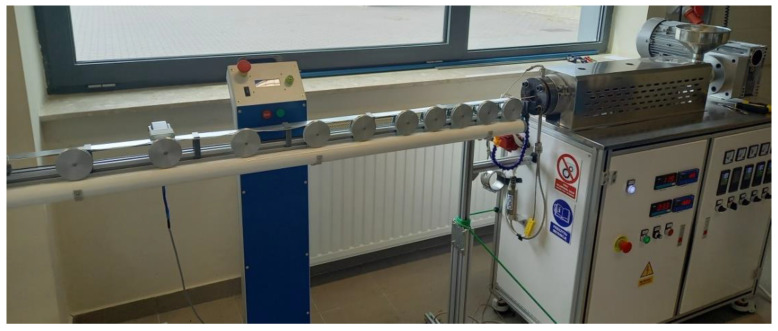
Technological line for the production of filaments.

Composites in the form of granules were obtained using a twin-screw extruder. The process was performed in the temperature range of 210–230 °C, at a screw rotational speed of 400 RPM, reaching an extrusion capacity of 4 kg/h. The granules were seasoned for 24 h at room temperature. Filaments were produced from composites using the parameters in Table 2, using the technological line shown in Figure 9. Dumbbell-shaped samples for testing the strength and structural characteristics were manufactured using the Prusa i3 MK3. The process parameters are presented in Table 2.

Based on the produced research samples, the research process was conducted, which consisted of *mass-melt flow rate* (MFR), thermogravimetric analysis of modified hydroxyapatite, Rockwell hardness, Charpy Impact Strength, and determination of static tensile strength. Determination of melt index according to ISO 1133:2011 [52] was performed using a DYNISCO 4781 apparatus (*Kayeness Inc.*, *Honey Brook*, *PA*). The test parameters are presented in Table 3.

Thermal analysis of the obtained materials was performed using the thermogravimetric analysis TGA method, consisting of measuring the change in the mass of the sample depending on the temperature. For the determination, a TGA/DSC 1 apparatus (*METTLER Toledo*, *Schwerzenbach*, *Switzerland*) was used, and the test was performed under a nitrogen atmosphere. For this purpose, about 5 mg of the sample was heated from room temperature to 600 °C on platinum crucibles with a heating rate of 10 °C/min. The hardness test consisted of pressing a ball-shaped indenter made of steel with a standardized diameter into the test sample. The indenters were each subjected to a total load of 358 N. The test was conducted using a Zwick/Roell 3106 hardness tester (*Zwick/Roell*, *Ulm*, *Germany*), following the recommendations of ISO 2039-1:2001 [53]. For each composition, the result was the arithmetic mean of 10 measurements. The impact strength was determined using a PSW4J type apparatus (*Gerhard Zorn*, *Berlin*, *Germany*). A 1 J impact energy hammer was used. The test was conducted following the PN-EN ISO 179-1:2010 [54] standard in a flat, edge arrangement, with a notch depth of 1 mm. The obtained composites in standardized samples were tested to determine their static tensile strength. The determination was conducted on the INSTRON 5967 testing machine (*Instron*, *Norwood*, *MS*, *USA*) at a temperature of 23 °C. The machine was controlled using the Bluehill 3 program. The tensile speed was 1 mm/min. Based on the obtained research, a decision was made to use unfilled PLA for the manufactured anatomical structure.

**Figure 10:**
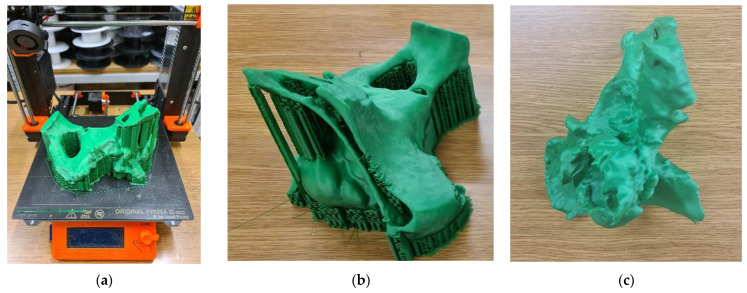
Model of the anatomical structure: (**a**) Situated within the working space of 3D printer; (**b**) After detaching from the working platform; and (**c**) Following the removal of the supporting structure.

Figure 10a shows an exemplary model of the anatomical structure manufactured using unfilled PLA material, located in the working space of the Prusa i3 MK3 device, together with the supporting structure, after detaching from the working platform (Figure 10b) and after removing the supporting structure (Figure 10c).

**Figure 11:**
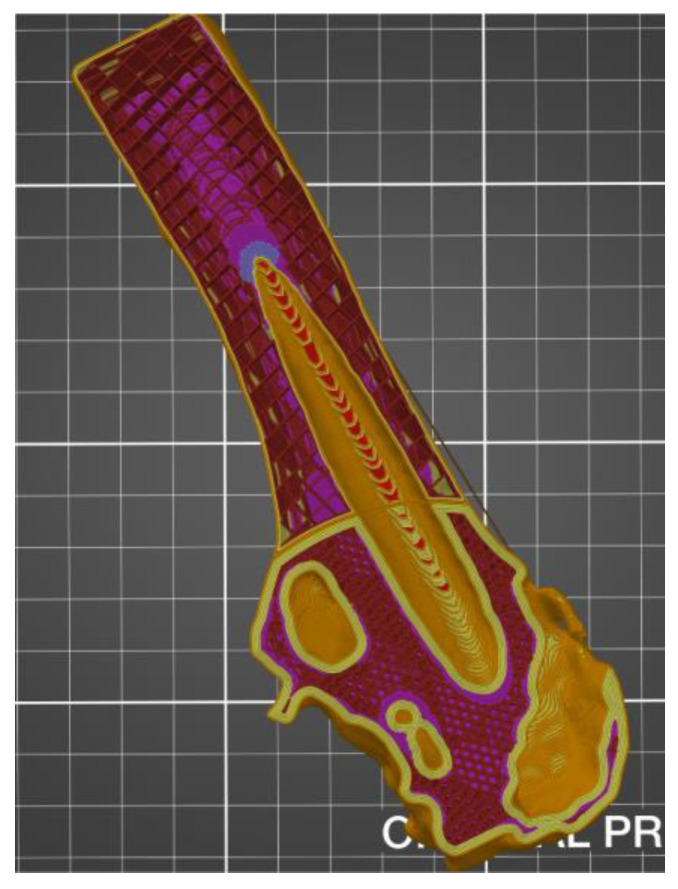
View of the anatomical model of the part of the femoral bone in the working space of the PrusaSlicer silver with the added modifier rendering it possible to change selected parameters.

**Figure 12:**
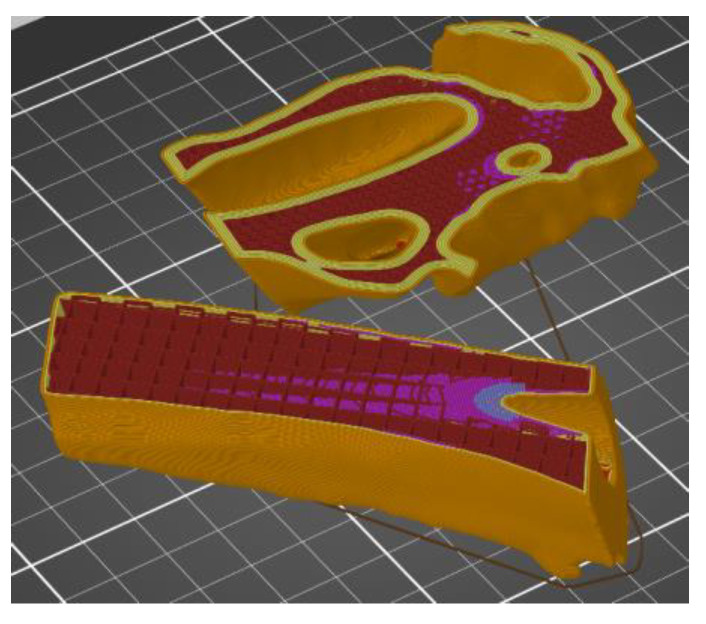
View of the anatomical model of the part of the bone in the working space of the software.

The additive FFF method used in the presented work and the device mentioned above allowed us to reduce costs and production time. It was possible thanks to taking into account a lot of factors during the printing. One of them was the division of the model into smaller parts—this procedure allowed for the printing of model fragments on separate devices with different production parameters. Additionally, when printing the models in the PrusaSlicer software, the height of the applied layer, density, filling, and the number of external contours were changed. However, it was noticed that the mentioned parameters in the case of adding modifiers for the entire model (Figure 11) or divided into fragments (Figure 12) might differ in the areas selected by the user. Figure 11 shows the use of the modifier for an undivided object representing the entire head and the shaft of the femur. In the case of the geometry presented in this way, the modifier allowed us to change the density, type of filling, number of strokes, and the type of the supporting structure. However, it was not possible to change the height of the superimposed print layer. In the case of the division of the femur into two separate parts representing the head and shaft of the femur (Figure 11), it was possible to change the abovementioned parameters, as well as the height of the print layer.

**Figure 13:**
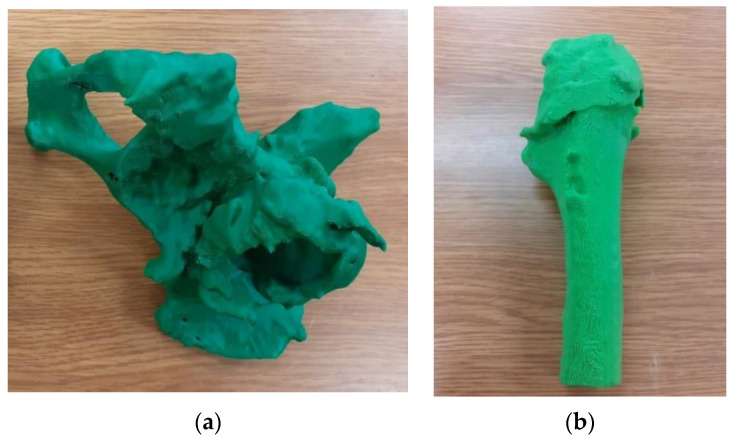
Model of the anatomical structure: (**a**) Part of the bone of the pelvis with the bone cement, and (**b**) the upper part of the femoral bone.

For testing both solutions, 3D prints of the hip joint model with a part of the femur for Patient No. 7 were performed in two ways. The first one consisted of making a single bone cement model together with the pelvic bone (Figure 13a) and the upper part of the femur (Figure 13b).

**Figure 14:**
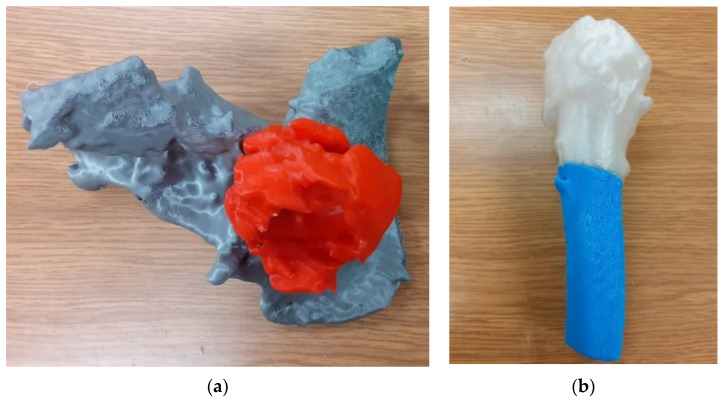
Model of the anatomical structure: (**a**) Bone cement (red color) and part of the bone of the pelvis (gray color) and (**b**) the femoral head (white color) and part of the femoral bone shaft (blue color).

The second method was to divide both models into two separate parts reflecting the bone cement and pelvic bone (Figure 14a) and the head and shaft of the femur (Figure 14b), according to the procedure presented in this publication. In the case of dividing the model into parts, an increase in the accuracy of structures was observed. Additionally, the amount of material used during 3D printing was reduced.

**Figure 15:**
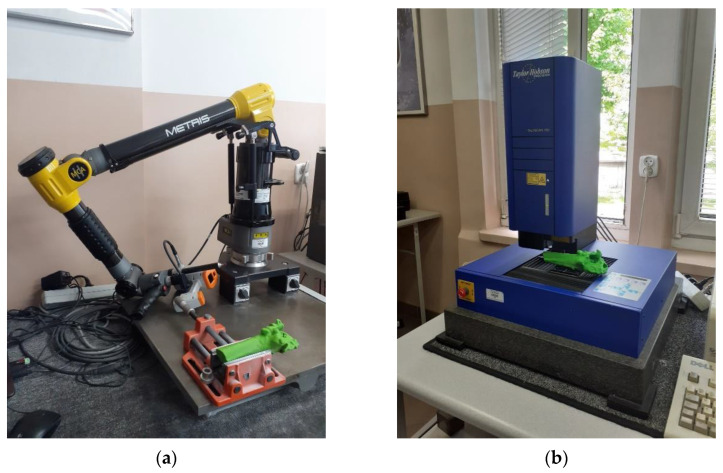
Measuring systems: (**a**) MCA-II measuring arm with the MMD×100 laser head, and (**b**) 3D Talyscan 150 profilometer.

The accuracy of the model geometry was verified using the MCA-II measuring arm with the MMDx100 laser head (*Nikon Metrology*, *Leuven*, *Belgium*) (Figure 15a). The accuracy of the coordinate measuring arm was checked against the ASME B89.4.22 standard [55] and a procedure presented in the publication [56]. Measurements of the printed models were conducted in two mountings using the highest resolution the system could use, which was 0.01 mm. In the digitization process, measurement data were obtained using a set of points with the coordinates x, y, and z representing the measured surface.

Surface roughness measurements were conducted using a 3D Talyscan 150 profilometer (*Taylor Hobson*, *Leicester*, *UK*) (Figure 15b), using the contact method. According to the procedure presented in the publication [57], the system accuracy assessment procedure was performed on the PGN-3 standard. In evaluating the roughness parameters of the printed models, they adopted the highest possible resolution of 5 μm and the lowest available measurement speed of 2000 µm/s. A single measured area was 4 mm × 4 mm. On each tested model, five measurements of the selected areas in each of its planes were made to average the obtained roughness results, presented in Tables 10 and 11.

## 3. Results

### 3.1. Results of Tests of PLA-Based Polymer Materials

Based on the produced dumbbell-shaped samples, the research process was conducted, which consisted of *mass melt flow rate* (MFR), thermogravimetric analysis of modified hydroxyapatite, Rockwell hardness, Charpy Impact Strength, and determination of static tensile strength. The results of the mass-melt flow rate test for all materials are shown in Table 4. In each case, the result is the mean value for three measurements.

**Figure 16:**
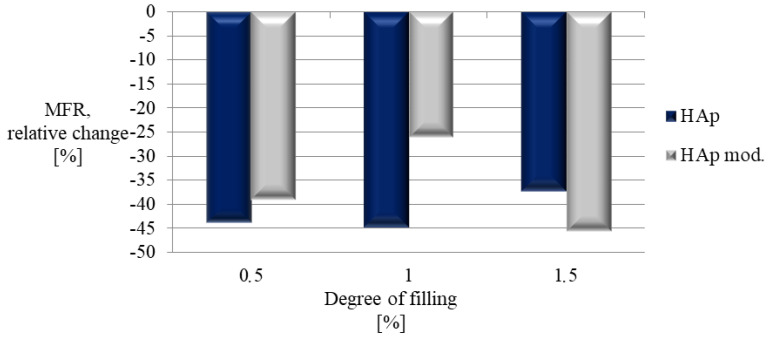
Relative change in mass-melt flow rate.

A significant reduction in the mass-melt flow rate with the unfilled material was observed for each composite. However, all samples were obtained using the parameters recommended by the manufacturer for unfilled PLA, without the need to raise the extrusion temperatures or the working table. Figure 16 shows the relative change in MFR for all composites with the unfilled material. The lowest decrease (26%) was recorded for the composite containing 1% of modified hydroxyapatite. At the same time, for the remaining cases, the values were similar, which shows that the modification of the filler does not have a significant impact on the mass flow rate of the composite produced with its participation.

The results of the thermogravimetric analysis are presented in Figure 4. The temperature of 2% weight loss, corresponding to the temperature of the loss of volatile compounds, is lower in the case of PLA than the value corresponding to the composites. The exception is composite IV, for which this value is close to T_2%_ for unfilled material. In the case of all composites, the 5% mass loss temperature turned out to be higher than the value determined for the unfilled material (Table 5). 

**Figure 17:**
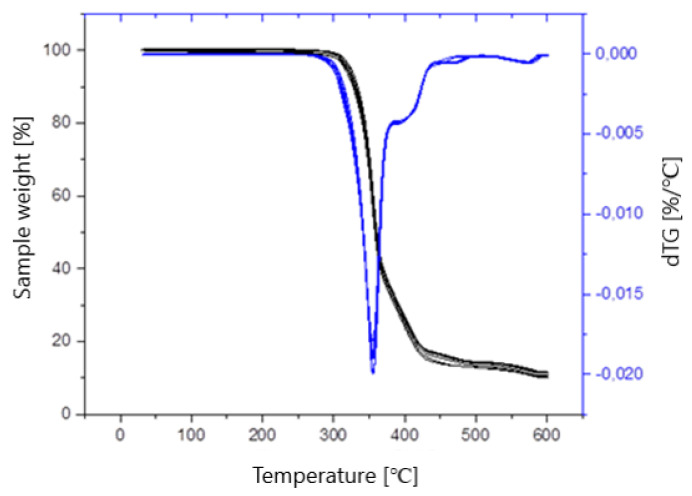
Thermogravimetric curve.

This means that the addition of both modified and unmodified hydroxyapatite slightly improves the material’s thermal resistance. The values of T_max_, i.e., the temperatures for which the value of the first derivative of the thermogravimetric curve reached the maximum, do not significantly differ, regardless of the degree of filling. The greatest percentage of weight loss corresponding to T_max_ was observed in the case of unfilled material. The residue after the test, R_600_, slightly differs between the tested samples and has a higher value for composites than for unfilled PLA, except for composite V. The graph (Figure 17) shows a thermogravimetric curve showing the changes in the mass of samples of the obtained composites and unfilled material, depending on the temperature (black) and its first derivative (blue). Based on the analysis of the chart, it was found that the material was decomposed in one step, as evidenced by one maximum on the dTG curve.

The results of the Rockwell hardness tests are presented in Table 6. In each case, the result is the arithmetic mean of 10 measurements.

**Figure 18:**
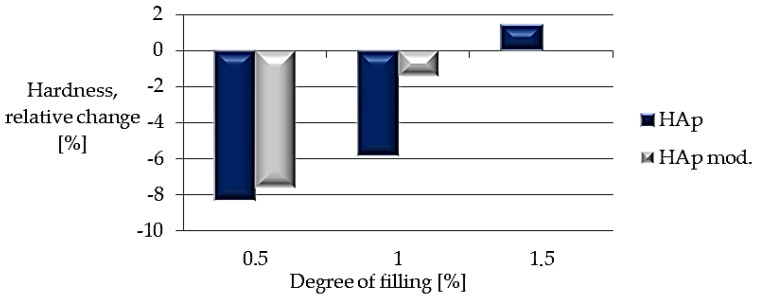
Relative change in hardness.

Based on the analysis of the results (Figure 18), no significant effect of the filler used was found. The greatest decrease in hardness (8.32%) was noted for the lowest filling, 0.5%. In comparison, for composites containing 1.5% of the filler, regardless of the filler’s use or lack of modification, the hardness of the material was close to the hardness value for the unfilled material.

The results of Charpy impact toughness are summarized in Table 7. The given values are arithmetic means for five samples, composites, and PLA.

**Figure 19:**
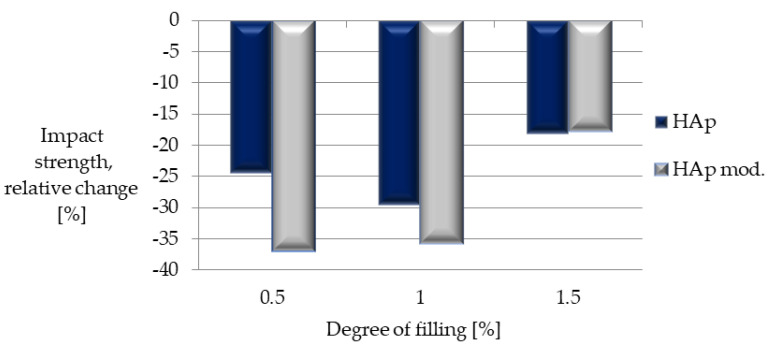
Relative change in impact strength.

A significant decrease in cracking resistance under dynamic loading was observed for all composites (Figure 19). For two of the three levels of filling tested (0.5 and 1%), the percentage of impact toughness decrease turned out to be higher for composites containing the modified filler.

The tensile strength of the obtained composites was also determined by measuring certain parameters, such as Young’s modulus, stress at the break of the sample, and percentage strain. The results, representing the mean values of the five samples, are summarized in Table 8.

**Figure 20:**
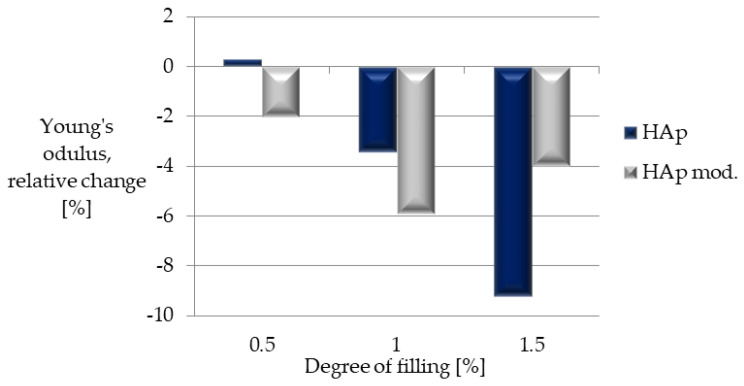
Relative change in Young’s modulus with respect to unfilled PLA.

The analysis of Figure 20, which shows the relative change of Young’s modulus of the obtained composites to PLA, confirmed that the addition of HAp slightly reduces the mechanical strength of the obtained composites. Additionally, the highest value of the relative change in Young’s modulus was recorded for the composite containing 1.5% of unmodified hydroxyapatite.

**Figure 21:**
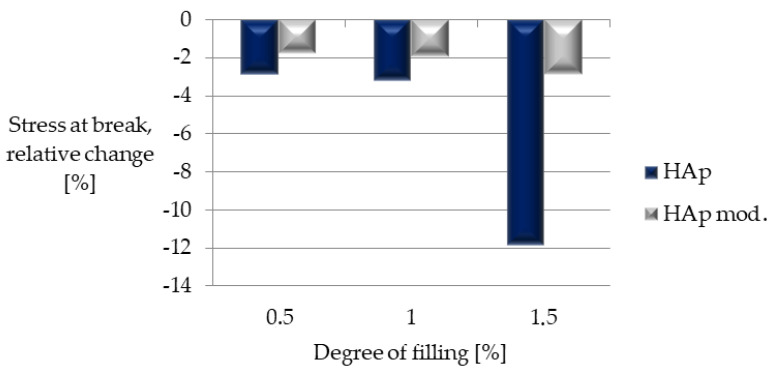
Relative change in breaking stress with respect to unfilled PLA.

The stress at the moment of rupture of the sample also decreased in the case of all composites (Figure 21). However, for unmodified hydroxyapatite, it increased with the increase in its concentration; while, in the case of samples filled with the modified filler, this parameter remained at a similar level.

**Figure 22:**
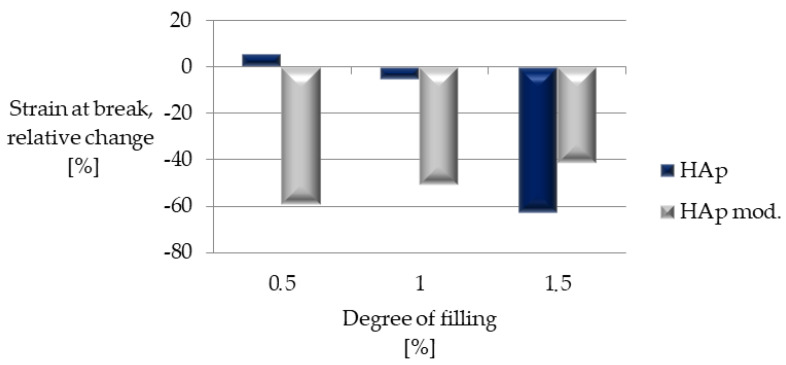
Relative change in strain at break with respect to unfilled PLA.

It was also analyzed how the elongation at break changes for individual composites (Figure 22). In the case of materials filled with modified hydroxyapatite for all filling levels, a significant decrease in the value of this parameter was observed (ranging between 40 and 60% for the unfilled material). In the case of modified hydroxyapatite for two lower filling degrees, elongation values similar to those of unfilled PLA were achieved. In the case of the highest filling degree, the most significant decrease in elongation was reached among all the tested materials.

Filling PLA with both modified and unmodified hydroxyapatite did not significantly affect the hardness and Young’s modulus of the obtained materials. The tested impact strength deteriorated for each of the composites. A minor decrease was recorded for composites filled with 1.5% HAp and 1.5% HAp mod. There was also a significant decrease in MFR for all materials, but it did not affect the parameters of the 3D-printing process. Filling PLA with hydroxyapatite, both modified and unmodified, slightly improved the material’s thermal resistance. As the analyzed test results of the obtained composites only differed somewhat from unfilled PLA, unfilled PLA was used for the manufactured anatomical structure. 

### 3.2. Results of Tested Procedure

**Figure 23:**
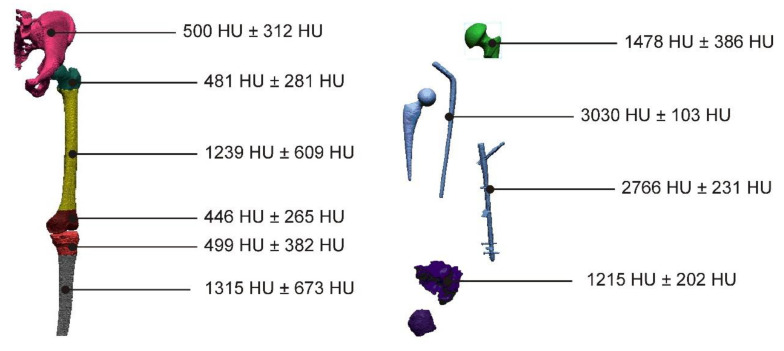
Obtained values (average value ± standard deviation) of the segmentation thresholds for anatomical structures, implants, and also the bone cement, within the areas of the hip joint and also the knee joint.

Thanks to the applied procedure, the segmentation thresholds were averaged on the basis of 10 cases, which allowed for a more accurate development of the numerical models of anatomical structures, implants, and bone cement (Figure 23).

In the process of 3D-printing anatomical structures, unfilled PLA material was used. Table 9 includes additional 3D-print parameters compared to Table 1, and information related to the printing time and costs incurred.

The first variant (Variant 1) was the continuous 3D printing of the anatomical model without division with the same parameters throughout its volume. In the second variant (Variant 2), a modifier was introduced in selected parts of the bone, allowing it to change the selected parameters. The remaining part of the bone was printed with parameters allowing us to shorten the production time of the selected anatomical structure. The last variant (Variant 3) was the division of anatomical structures allowing for 3D printing with different production parameters and on two devices. By analyzing the obtained results, it can clearly be stated that Variant 1 is the least favorable in terms of time because, even if the total time exceeds the time of manufacturing two separate parts of anatomical structures, one after another, it should be remembered that the paper assumes the possibility of manufacturing models in an additive method at the same time on multiple devices. In the case of the hip joint, Variant 3 was the most economical both in the anatomical structure of the acetabulum area with bone cement and in the upper part of the femur. Even though the difference between Variants 2 and 3 for the upper femur was negligible, it was related to the volume of the model. Therefore, along with the reduction in mainly filling, the time decreased, and the material consumption decreased, which translates directly into the cost of manufacturing a 3D model. In the case of the knee joint, it is not so clear-cut because the costs of Variants 2 and 3 of the upper part of the tibia are identical, but for lower than the costs of Variant 1. However, in the case of the lower part of the femur, Variant 2 was the most economical, and Variant 3 the least. It is mainly because the knee joint structures have been made in the form of a shell where the filling is not very important for the economic aspect, and the main factor is the time of manufacturing the 3D model. To reduce the time in selected variants, you can, for example, try to further divide the selected structure or change the arrangement in the working space of a 3D printer.

The process of verifying the manufacturing model’s accuracy was performed on the data obtained from the MCA-II measuring arm system with the MMDx100 laser head. The adjustment of the nominal model obtained at the RE/CAD design stage and the reference model created at the measurement stage was conducted using the best fit method with an accuracy of 0.001 mm. The final results for the printed models are presented in Table 10 and Table 11. In the case of data obtained from the Talyscan 150 3D profilometer, it was analyzed in the MountainsMap software. In determining the surface roughness parameters, the shape errors were first removed. Then, to separate the long-wave components, a profile filter λc = 0.8 mm was used. The value of the sampling length was determined based on the ISO 4288 standard [58]. The final results representing the stereometric parameters determined based on the ISO 25178-2 [59] standard are presented in Table 10 and Table 11.

Based on the printed models, surgical procedures were planned, the effects of which are presented in this publication on the example of 3 out of 10 analyzed patients.

**Figure 24:**
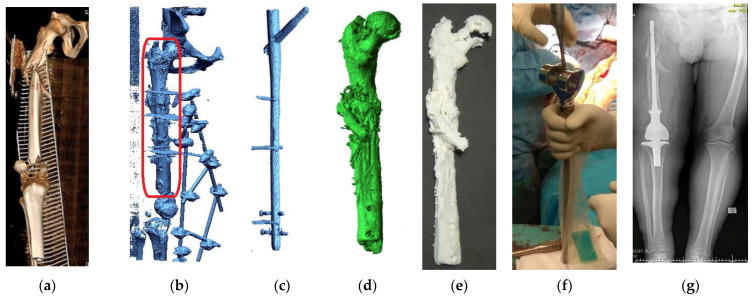
Process of planning the procedure as exemplified by Patient No. 2: (**a**) Diagnostics following an accident; (**b**) Reconstructing volumetric data, together with the marked area to be printed; (**c**) The digital reconstruction of the traction pin; (**d**) The digital reconstruction of the femoral bone; (**e**) The printed model of the femoral bone; (**f**) Prosthesis of the knee joint; and (**g**) Diagnostics following the performed procedure.

Patient no. 2 concerns an example of treatment of a complex transport accident of a 40-year-old man, 180 cm tall, and weighing 120 kg. As a result of a motorcycle accident, he suffered multi-organ injuries, including numerous lacerations and burn injuries to the lower limbs, the patellar attachment of the right quadriceps muscle was torn, and an open (IIIA according to G-A) fracture of the right femur (32-C3 IO3 according to AO) with a defect in the distal end of the femur (Figure 24a). On the day of the injury, the external fixation of the femoral fracture was performed with the supplementation of the defect in the distal epiphysis with an intraoperative cement SPACER. Then, additional stabilization of the femoral shaft fracture was achieved with an intramedullary nail. The external stabilizer was removed to prepare for the final supply, leaving the orthosis. The last stage was preceded by planning on 3D bone models and software for the design of custom-made prostheses and numerous engineering consultations. Significant noises characterized the patient’s diagnostic data due to the occurrence of metallic structures stabilizing the fracture area (Figure 24b). Therefore, the DICOM data were processed by the procedure presented in the article. The intramedullary nail (Figure 24c) was digitally separated from the femur (Figure 24d). Then, the femur model was reconstructed, and the model was printed (Figure 24e). The 1:1 scale model was used to evaluate the bone union and calculate the implantation of an individually designed femoral stem in a specialist Zimmer Biomet company that produces high-class joint endoprostheses (Figure 24f). The patient’s case was consulted with many specialists in Poland and abroad. Ultimately, the knee was not stiffened to put on a resection prosthesis. Thanks to the models made using the 3D-printing technique, it was possible to visualize the pathological problem better, adjust the implant to the patient’s bone structures, and select the surgical process more precisely during the procedure. The use of 3D printing also allowed for the avoidance of intraoperative difficulties related to excessive periosteal reactions arising during the union of a multi-fragment bone fracture. In the last stage, after the treatment planning, the external stabilizer and the intramedullary nail were removed, with the simultaneous insertion of the prosthesis associated with an individually designed, extended custom-made femoral stem. Within three years, subsequent follow-up postoperative radiographs showed no signs of implant loosening (Figure 24g).

**Figure 25:**
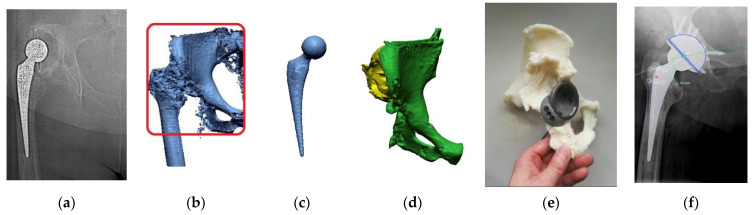
Process of planning the procedure as exemplified by Patient No. 7: (**a**) Diagnostics prior to the procedure; (**b**) Reconstruction of the volumetric data, together with the marked area to be printed; (**c**) The digital reconstruction of the endoprosthesis of the hip joint; (**d**) The digital reconstruction of the pelvis, together with the cement; (**e**) The printed model with adjusted implant; and (**f**) Diagnostics following the performed procedure.

Patient No. 7: a 74-year-old woman, weight 105 kg, height 165 cm, 12 years after the original and two years (2015) after revision of the right hip arthroplasty performed in another center, during which the acetabulum was placed in the wrong place, i.e., above the anatomical acetabulum (Figure 25a). As a result, the limb was shortened by about 7 cm, with gait failure and chronic pain syndrome. During the preparation for the revision procedure, CT was performed to assess the possibility of effective and safe placement of the implants. Due to the significant defects in the roof and the posterior wall of the acetabulum of the hip joint, it was decided to create a 3D model of the pelvis with a natural size to assess the possibility of correct and permanent seating of the prosthesis acetabulum. Due to the prosthesis and the bone cement in the analyzed area of the hip joint, it was necessary to apply the procedure presented in the article (Figure 25b). First, the endoprosthesis was digitally segmented (Figure 25c). In the next step, the bone cement was segmented along with the pelvis (Figure 25d). At the operator’s request, to facilitate intraoperative orientation, apart from the bone, the bone cement was also left on the pelvic model, located under the acetabular prosthesis. During the preparation for the procedure, thanks to the model, various positions of the implants were tested, and their most optimal setting was planned, including the place and location of the augmentation, the position of the cup, and the direction and length of the fixing screws (Figure 25e). It allowed both to limit the number of implants and obtain their most excellent stability. The time of the procedure itself was also shortened by avoiding the intraoperative search for a good position for the implants. Both the right rotation center of the hip joint and the length of the limb were restored. Eventually, the implants were healed, and the quality of the patient’s walk and life improved (Figure 25f).

**Figure 26:**
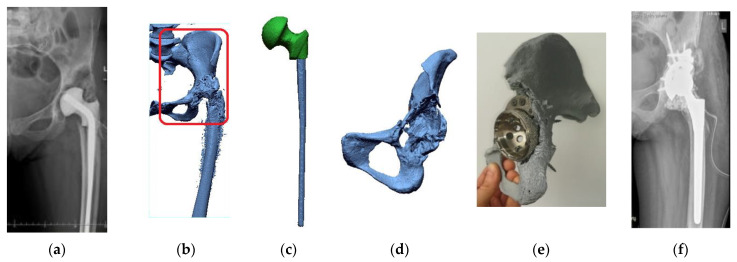
Process of planning the procedure as exemplified by Patient No. 10: (**a**) Diagnostics prior to the procedure; (**b**) Reconstructing volumetric data, together with the marked area to be printed; (**c**) The digital reconstruction of the endoprosthesis of the hip joint; (**d**) The digital reconstruction of the pelvis; (**e**) Printed model of the pelvis, together with the adjusted implant; and (**f**) Diagnostics following the performed procedure.

A female (Patient No. 10) was after primary arthroplasty due to degenerative changes in the hip joint during dysplasia and its revision in December 2019, due to loosening of the acetabulum and prosthesis stem. Due to the intraoperative suspicion of infection during the revision procedure, the patient was provided with a temporary cement implant that releases an antibiotic, the so-called SPACER (Figure 26a). Further diagnostic and laboratory tests were performed, while planning the following revision procedure to supply the patient. Due to the extensive destruction of the pelvic bones in the acetabulum area, an attempt was made to create a life-size 3D model of the pelvis. During the analysis and overall reconstruction of the DICOM data, the noise in the area where the endoprosthesis was located was visualized (Figure 26b). To accurately isolate the pelvis, the endoprosthesis was first digitally segmented (Figure 26c). Then, a digital model of the pelvis was recreated, which was later used for planning the procedure (Figure 26d). The prints served as anatomical models to consider various treatment options. After several trials with different implants, it was decided to use specific implants. Their position and the position and orientation of the fixing screws were planned (Figure 26e). On 24 March 2020, realloplasty was performed. The procedure consisted of cleaning the bottom of the acetabulum from scars, following it with an acetabular cutter, and supplementing and strengthening with allogeneic bone grafts to prepare the substrate for implant placement. Thanks to the 3D pelvis model, the type and position of the implants were known, so no time was wasted on the search for the best solutions, but the focus was on reconstructing their position as accurately as possible on the 3D model and stabilizing them with screws by their planned directions and lengths. As a result of the procedure, the right rotation center of the hip joint, the size of the limb, the original stability of the implants, and the full range of movements of the operated hip were restored (Figure 26f). Together with the radiological diagnostics performed, the printed models presented a faithful image of the operated area. They made it possible to plan and compare the possibilities of using various available implants. As a result, they enabled the use of a procedure that is less mutilating and spares more the preserved natural bone of the patient than the most commonly used and recommended for this type of extensive damage to the bone base, individualized prostheses requiring resection of a significant part of the pelvis “custom made” prostheses.

## 4. Discussion

The accuracy of the anatomical structure model is influenced, by the type of diagnostic system used [11,12], data processing methods (taking into account the process of segmentation and reconstruction of geometry) [16,17], and the selection of a method and optimization of the manufacturing process [56,60,61,62]. Nowadays, a lot of research is conducted to assess the accuracy of the execution of anatomical structures. However, there is no systematic procedure for infrequently damaged bone structures and those which contain metallic structures. Considering the literature review, the quality of DICOM data is assessed based on spatial and contrast resolution [12,13]. They depend on the type of diagnostic system and the level of artifacts (especially metallic ones). The low diagnostic quality of DICOM data also impedes the segmentation process [7,8]. Thanks to the methodology used in the article, taking into account the digital filtration and data interpolation, the spatial and contrast resolution of the data was increased. This process improved the segmentation process of bone structures, including those that occur within metallic structures. Various types of segmentation algorithms can be found in the literature [10]. However, the thresholding option is still the most frequently used segmentation method [9]. However, as with any technique, it has some limitations, mainly due to difficulties with the correct selection of segmentation thresholds. The failure to establish the appropriate threshold or thresholds for segmentation may change the shape and volume of the reconstructed anatomical structure [7,8]. Thanks to the development of averaged values of local segmentation thresholds in the publication, it is possible to more reliably reproduce the geometry of the anatomical structure and the implant. At this stage of reconstruction of numerical anatomical models, it is necessary to properly prepare the digital data. Unfortunately, the algorithms that allow the reconstruction of anatomical structures in a three-dimensional form (e.g., planar contour or voxel-based methods) have certain limitations that affect the quality of the obtained faceted structure [11]. The faceted structure with errors makes it much more difficult to print the model directly, which, at the same time, extends the data processing time [40,41,42]. Thanks to the developed procedure, it is possible to efficiently remove the most common errors of the faceted structure arising in model geometry reconstruction from DICOM data, thus increasing the accuracy by interfering with the settings of the angular deviation and the chord. In addition, in the process of 3D-printing models, care was taken to select parameters that increase the manufacturing accuracy related to the layer thickness, the orientation of the model in the printer space, and the type of material. In the case of large-sized objects, dividing the model into parts was used. Considering the current literature review, a rapid increase in polymeric materials in the additive manufacturing of anatomical structures [56,61], also made of PLA material, can be observed [56,61,63,64]. There are also studies on the accuracy of anatomical structures made of this material [56,61]. Considering the publications [56,61], the models made of PLA material are within the tolerance of ±0.18 mm. In the case of the method presented in the manuscript, among other divisions into parts of the models, better accuracy of models was obtained in the case of the proximal femur, femoral shaft, and tibia shaft with a tolerance of ± 0.1 mm. Apart from further increasing the accuracy of the geometry printing, the purpose of this procedure was to reduce the printing time and thus the manufacturing costs.

Thanks to the conclusion of a cooperation agreement, communication between the Department of Orthopaedics and Traumatology employees of the Provincial Clinical Hospital No. 2 in Rzeszów and the Rzeszów University of Technology was significantly improved. The obtained printouts of the models were used to plan surgical procedures. In the case of Patient No. 2, the printout of the model made it possible to assess the bone union and the spatial form of setting, and the strength of the callus connection of the multi-fragment fractures of the femoral shaft. Due to the lack of the distal femoral epiphysis, it was decided to insert a resection prosthesis with a custom-printed titanium extension of the femoral implant [65,66,67]. The situation with a coexisting shaft fracture at a distal femoral epiphyseal defect has not been described in the literature to date. Therefore, it required an innovative approach and an individual technical solution to this problem [68,69,70]. These actions allowed us, first of all, to shorten the time of immobilization and earlier commencement of rehabilitation to faster recovery of the flexion and extension movement functions in the knee joint. In the case of patients No. 7 and 10, due to the extensive loss of the acetabulum, an additional advantage of the printout was the ability to practice different versions and configurations of implants, which is essential in the case of such extensive pelvic defects, which give the operator virtually no margin of error. For this reason, such cases end up badly for the patient or require the use of special individual “custom made” implants. In addition to costs, they are also burdened with a high risk of complications and exhaust the options of surgical treatment, which is particularly important in young patients for whom good long-term functioning is vital. Thanks to the printed models in the operating room, it was possible to better visualize the anatomy visible in the operational area. In all the presented cases, the implants were successfully healed, improving the patient’s quality of life and giving them a chance for proper functioning. Thanks to the detailed, meticulous planning of the procedure, the type and position of the implants, and the length of their stabilizing screws, both the risk of failure and intraoperative complications were reduced. The time of procedures was shortened, and the need for mutilating procedures using post-resection prostheses was reduced. In the case of non-standard pathologies, e.g., covering extensive bone tissue defects, it is necessary to print a model showing the type and size of the pathology. The use of models allows for avoiding many unnecessary operational errors. However, it is required to conduct further research processes, which allow for the development of biodegradable materials [71,72,73] and reflect the structure and strength of human bone as accurately as possible [74,75], because the models presented in the article had a susceptibility to cutting and milling that was different from natural bone. When planning the procedure, the models presented in the article were made of classic polymer materials, for which it was difficult to perform efficient cuts and drilling due to their plastic deformation. On the other hand, the hybrid materials planned for development will have similar hardness and allow, as mentioned above, to obtain a structure identical to the natural human bone (hard and spongy part). Such obtained parameters of the artificial bone will enable the analysis of the course of the operation and non-invasive training as close to reality as possible. 

## 5. Conclusions

Thanks to the presented patented procedure, it is possible to more accurately reconstruct the geometry of anatomical structures and when metallic structures appear in the diagnostic image within the bone tissues. The models based on the presented procedure allowed for detailed and meticulous treatment planning. The type of implants, their direction, and attachment method have been precisely planned, thus avoiding mutilating procedures. However, continuous improvement of the presented technique is required. The aim of our further research will be the acceleration of the DICOM data processing itself and the printing of models. We will also focus on testing other materials to make the models printed from them closer to structural mapping and mechanical imitation of natural human bone. The models based on the presented procedure allowed for the successful connection of the final implants with the bone, recreating patients’ motor function and giving them a chance to function correctly in society.

The adaptation of numerical procedures used industrially in mechanical engineering to the planning of medical procedures and increasing the accuracy of medical models produced with additive methods allows us to shorten the time of preparation of the operation and often also shorten the time of the surgical process itself. Medicine and medical engineering can successfully apply the procedures based on the Industry 4.0 philosophy. An essential element of the network integration of medical and technological data is the acceleration of decision-making processes in the medical diagnostic process may ultimately reduce operating times and patient treatment costs.

## 6. Patents

Resulting from the presented procedure in this manuscript is the granting of a patent “A method of producing anatomical models” by Patent Office of the Republic of Poland. Patent number: 239300.

## Figures and Tables

**Table 1:** Composition of the obtained composites.

Sample	PLA Content [%]	HAp Content [%]	HAp Mod. Content [%]
Composite I	99.5	0.5	0.0
Composite II	99.0	1.0	0.0
Composite III	98.5	1.5	0.0
Composite IV	99.5	0.0	0.5
Composite V	99.0	0.0	1.0
Composite VI	98.5	0.0	1.5

**Table 2:** Parameters of filament manufacturing and 3D-printing process.

Filament Manufacturing Process		3D-Printing Process	
Parameters	Value	Parameters	Value
Temperature	190–220 °C	Nozzle diameter	0.4 mm
Screw rotational speed	120 RPM	Layer height	0.2 mm
Extraction speed	110 mm/s	Infill percentage	100%
Filament diameter	1.75 ± 0.05 mm	Infill pattern	Rectilinear ± 45°
		Extrusion temperature	220 °C
		Bed temperature	40 °C
		Printing speed	70 mm/s

**Table 3:** Parameters of the MFR test.

Parameter	Value
Preload	1.1 kg
Basic load	2.16 kg
Temperature	220 °C
Time	240 s
Sample cut-off time	5 s

**Table 4:** Parameters of the MFR test.

Sample	MFR [g/10 min]
Composite I	24.36 ± 0.03
Composite II	24.07 ± 0.04
Composite III	27.24 ± 0.12
Composite IV	26.45 ± 0.02
Composite V	32.09 ± 0.05
Composite VI	23.64 ± 0.00
PLA	43.45 ± 0.04

**Table 5:** Thermogravimetric analysis results.

Sample	T_2%_ [°C]	T_5%_ [°C]	T_max_ [°C]	m_max_ [%]	R_600_ [%]
Composite I	307.63	319.59	355.51	40.99	10.35
Composite II	312.50	323.97	355.17	39.13	10.71
Composite III	310.54	321.72	355.57	40.11	11.74
Composite IV	300.52	317.29	355.38	41.62	10.09
Composite V	306.37	318.12	354.77	40.67	11.88
Composite VI	312.99	324.01	355.39	38.92	11.77
PLA	300.69	315.40	354.17	43.22	10.18

**Table 6:** Rockwell hardness test results.

Sample	Hardness [N/mm^2^]
Composite I	31.1 ± 3.9
Composite II	31.9 ± 3.9
Composite III	34.4 ± 4.0
Composite IV	31.3 ± 3.6
Composite V	33.4 ± 2.7
Composite VI	33.9 ± 4.0
PLA	33.9 ± 4.2

**Table 7:** Charpy impact test results.

Sample	Impact Strength [kJ/m^2^]
Composite I	4.99 ± 0.57
Composite II	466 ± 0.48
Composite III	5.41 ± 1.55
Composite IV	4.17 ± 0.62
Composite V	4.25 ± 0.53
Composite VI	5.44 ± 1.16
PLA	6.62 ± 0.91

**Table 8:** Static tensile strength test results.

Sample	Stress at Break, [MPa]	Young’s Modulus, [MPa]	Strain at Break [%]
Composite I	32.4 ± 0.5	1409.0 ± 21.3	18.9 ± 3.8
Composite II	32.3 ± 0.5	1356.8 ± 24.0	16.9 ± 6.1
Composite III	29.4 ± 0.2	1275.7 ± 25.5	6.7 ± 4.0
Composite IV	32.7 ± 0.2	1376.4 ± 46.9	7.3 ± 4.7
Composite V	32.7 ± 0.4	1322.2 ± 48.1	8.8 ± 6.2
Composite VI	32.4 ± 1.0	1348.9 ± 39.3	10.5 ± 4.9
PLA	33.3 ± 0.8	1405.1 ± 54.7	17.9 ± 9.3

**Table 9:** Additional parameters and estimation of costs and time for 3D printing.

Region	3D-Printing Option	Additional 3D-Printing Parameters		Visualization	Time of 3D Printing [Days:Hours:Minutes]	Cost of 3D-Printing Model [USD]
		Number of Contours	Infill Percentage [%]			
**Acetabulum** **area**	Acetabulum (without added modifier)—**variant 1**	5	80	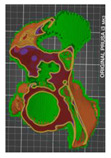	02:00:44	329.24
	Acetabulum (with added modifier)—**variant 2**	2 (modifier)	15 (modifier)	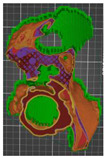	01:13:29	247.39
		5	80			
	Acetabulum (without added modifier)—**variant 3**	2	15	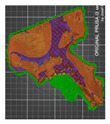	00:23:21	154.11
	Bone cement (without added modifier)—**variant 3**	5	80	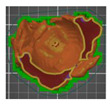	00:12:27	82.17
**Femur bone area—upper part**	Femur bone— (without added modifier)—**variant 1**	5	80	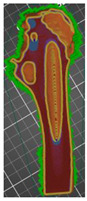	00:15:21	101.31
	Femur bone— (with added modifier)—**variant 2**	2 (modifier)	15 (modifier)	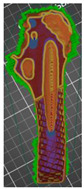	00:12:46	84.26
		5	80			
	Proximal femur (without added modifier)—**variant 3**	5	80	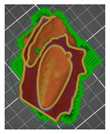	00:09:40	63.8
	Femoral shaft (without added modifier)—**variant 3**	2	15	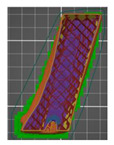	00:03:04	20.24
**Femur bone area—lower part**	Femur bone— (without added modifier)—**variant 1**	5	80	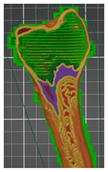	00:09:11	60.61
	Femur bone— (with added modifier)—**variant 2**	2 (modifier)	15 (modifier)	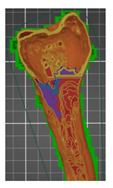	00:07:27	49.17
		5	80			
	Distal femur (without added modifier)—**variant 3**	5	80	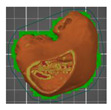	00:06:05	40.15
	Femoral shaft (without added modifier)—**variant 3**	2	15	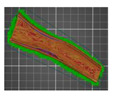	00:04:02	26.62
**Tibia bone area—upper part**	Tibia bone— (without added modifier)—**variant 1**	5	80	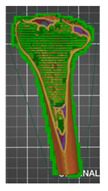	00:11:55	78.65
	Tibia bone— (with added modifier)—**variant 2**	2 (modifier)	15 (modifier)	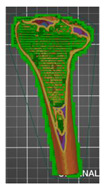	00:09:24	62.04
		5	80			
	Proximal tibia (without added modifier)—**variant 3**	5	80	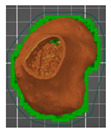	00:06:35	43.45
	Tibia shaft (without added modifier)—**variant 3**	2	15	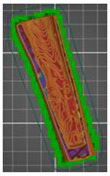	00:02:49	18.59

**Table 10:** Statistical parameters—without using a procedure.

Type of Analysis	Parameters	Acetabulum	Femur Bone	Tibia Bone
Accuracy of geometry	Mean deviation (ӯ) [mm]	−0.012	0.008	0.020
	Standard deviation (σ) [mm]	0.180	0.193	0.178
Surface roughness	Root-mean-square height (Sq) [μm]	7.430	7.080	6.800
	Arithmetical mean height (Sa) [μm]	6.020	5.800	5.930
	Maximum pit height (Sv) [μm]	14.530	20.750	16.230
	Maximum peak height (Sp) [μm]	10.540	9.230	8.320

**Table 11:** Statistical parameters—using a procedure.

Type of Analysis	Parameters	Acetabulum Area		Femur Bone Area			Tibia Bone Area	
		Acetabulum	Bone Cement	Proximal Femur	Femoral Shaft	Distal Femur	Proximal Tibia	Tibia Shaft
Accuracy of geometry	Mean deviation (ӯ) [mm]	0.010	0.023	0.004	−0.008	0.007	0.015	−0.002
	Standard deviation (σ) [mm]	0.124	0.152	0.102	0.105	0.162	0.142	0.092
Surface roughness	Root-mean-square height (Sq) [μm]	5.800	6.020	5.580	4.380	5.450	5.920	5.400
	Arithmetical mean height (Sa) [μm]	5.060	5.720	4.010	3.490	4.600	5.420	5.230
	Maximum pit height (Sv) [μm]	12.010	13.020	14.820	13.650	12.040	15.200	12.020
	Maximum peak height (Sp) [μm]	9.300	10.230	14.830	7.100	8.030	7.900	7.430

## Data Availability

Not applicable.
